# Advancements in the Evolution of Engineering Characteristics and Reinforcement Technologies for Subgrade Silt

**DOI:** 10.3390/ma16216965

**Published:** 2023-10-30

**Authors:** Xiaoyan Liu, Jinpeng Zhao, Lulu Liu

**Affiliations:** 1 School of Mechanics and Civil Engineering, China University of Mining and Technology, Xuzhou 221116, China; luckyliuxiaoyan@cumt.edu.cn; 2 School of Highway, Chang’an University, Xi’an 710064, China; peng@8260.chd.edu.cn; 3 State Key Laboratory for Geomechanics and Deep Underground Engineering, China University of Mining and Technology, Xuzhou 221116, China

**Keywords:** silt, recycled polyester fiber, subgrade reinforcement, fiber, constitutive model

## Abstract

Technical challenges associated with the treatment of silt subgrades frequently arise in coastal and river delta areas. Given the importance of environmental sustainability, the selection of efficient, cost-effective, and eco-friendly techniques for silt subgrade stabilization is paramount. While recycled polyester fibers primarily sourced from discarded polyester bottles have not yet been systematically employed in silt subgrade reinforcement, their potential is considerable. This paper offers a comprehensive review of the existing literature on the microstructural, physicochemical, and mechanical properties of silt, summarizing prior advancements in silt stabilization methodologies. Building upon this foundation, we introduce a novel approach utilizing recycled polyester fibers for silt subgrade improvement, outlining both its application prospects and challenges, which require further investigation. The findings of this study serve as a robust scientific foundation for the broader adoption and engineering implementation of this technology.

## 1. Introduction

In transportation engineering projects, silt subgrades are commonly encountered. Silt is academically defined as a fine-grained soil with a particle size greater than 0.075 mm comprising no more than 50% of the total mass, and a plasticity index of Ip ≤ 10 [1]. Notably, silt is prone to dust generation when dry and tends to liquefy upon contact with water, making it challenging to compact. Consequently, its suitability as a subgrade fill material is suboptimal. Under vehicular loading conditions, silt can readily cause pavement deformation, cracking, and instability [2,3]. Therefore, the direct usage of silt in roadbeds is inadvisable, as it necessitates pre-treatment via targeted technical measures. Advancements in silt reinforcement techniques hold significant theoretical implications for efficient silt resource utilization, and are urgently needed to foster sustainable practices in the geotechnical engineering sector [4].

The enhancement of silt properties through reinforcement techniques is a focal point in contemporary international research within the field of geotechnical engineering [5]. Existing studies demonstrate that the mechanical characteristics of silt can be substantially improved through the incorporation of inorganic materials such as cement, lime, and fly ash [6]. In comparison to non-reinforced soil, the augmented soil exhibits noticeable improvements across various engineering performance metrics [7]. Specifically, this improvement is manifest in enhanced soil mechanical properties, including an increase in both the internal friction angle and cohesion, as well as a bolstered shear strength, which, in turn, significantly elevates the soil’s bearing capacity. Additionally, the freeze–thaw resistance and water stability of the soil are improved, thereby enhancing its overall durability. As a result of these multifaceted advantages, soil reinforcement techniques have found extensive application in road engineering projects, yielding substantial social and economic benefits.

Situated between sandy and cohesive soils, silt exhibits engineering properties that diverge significantly from both types. The investigation of silt presents particular challenges: not only is the sample easily perturbed, but its reference values are influenced by a myriad of factors including soil type, stress history, and both pre- and post-test sample preparation. Consequently, the academic community faces considerable uncertainty and difficulty in evaluating silt’s engineering properties in comparison to those of sandy and cohesive soils. This paper comprehensively synthesizes prior research on silt, spanning its microscopic attributes, physical–chemical properties, and mechanical characteristics, as well as methods for its improvement. It further proposes the promising application of recycled polyester fiber for current silt soil enhancement, identifying key challenges that must be addressed. This provides a substantive scientific foundation for the further development and engineering application of this technique.

## 2. Micro Characteristics

Since the inception of soil mechanics in the early 20th century, spearheaded by its founding figures, scholarly attention has increasingly focused on the study of soil microstructures. One pivotal contribution was the introduction of the clay honeycomb structure, a conceptual breakthrough that has facilitated subsequent research into soil microarchitecture. Through a comprehensive set of laboratory tests—comprising compaction, permeability, and triaxial tests, as well as microstructural analyses—Wu et al. [8] scrutinized the mechanical properties of silt when employed as a subgrade filler in highway construction. Their study offers a comparative analysis of the dry density attributes of silt under varying levels of compaction energy. Additionally, they examine the relationship between compactness and void ratio, delve into the compaction performance across diverse gradations of silt, and assess both the permeability and mechanical attributes that are dependent on differing degrees of compaction. Their findings also include a detailed account of the microstructural arrangements of silt under various compaction conditions, providing a well-rounded exploration of both its macroscopic and microscopic characteristics.

In their seminal study, Delage et al. [9] employed both qualitative and quantitative methodologies to explore the microstructure of compacted silt. Utilizing techniques such as scanning electron microscopy and mercury intrusion porosimetry, they conducted static compaction tests on samples characterized by three distinct moisture levels. Their research reveals that wet samples manifest a matrix-like structure, wherein clay elements fill voids and adhere to silt particles. In contrast, the drier samples displayed a skeletal architecture composed of silt particle aggregates, which are interconnected via clay bridges. Additionally, the study provides a nuanced account of the distribution of water and air within the microstructure of compacted silt under these diverse compaction conditions.

In a comprehensive study, Yamamuro et al. [10] executed undrained triaxial compression tests on Nevada sand samples comprising 20% non-plastic silt. Various deposition methods were employed, including mud deposition, water deposition, air precipitation, mixed dry deposition, and dry funnel deposition. The team utilized scanning electron microscopy (SEM) to investigate the impact of both deposition techniques and silt content on particle contact structure. Additionally, they developed a computational program designed to quantify microstructural features based on the stability of grain-to-grain contacts. In a related study, the microstructural characteristics of unsaturated flaky clay was examined using both SEM and mercury intrusion porosimetry [11]. Their analysis focused primarily on clay microstructures, offering a quantitative evaluation of how the characteristics change with variations in compaction pressure during both loading and unloading cycles. Moreover, Guo [12] employed Matlab to generate three-dimensional visual representations of clay microstructures, further enriching the field’s understanding of these complex materials.

**Figure 1:**
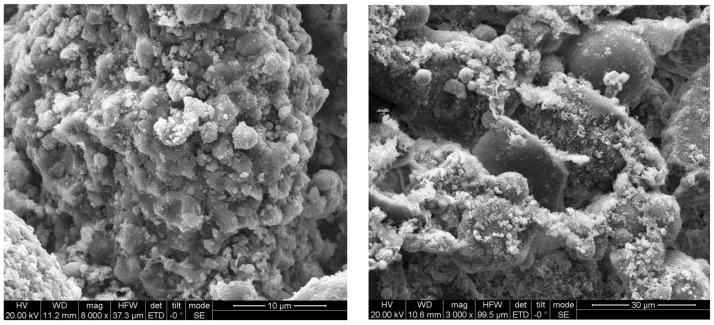
SEM image of silt.

The microstructure of silt (see Figure 1) plays a pivotal role in the liquefaction of silty soil. Liquefaction refers to when, through the repeated action of an external force, the soil is compressed and the internal void is reduced, there is an increase in water pressure in the void. When the water pressure rises to a level that exceeds the external pressure in the soil, plus the water cannot be discharged from the ground, liquefaction damage occurs. Two primary mechanisms have been identified for this phenomenon [13]. The first mechanism is thixotropy, the property that the strength of the soil gradually recovers after the structure of the soil is disturbed, resulting in a decrease in strength, but as the standing time increases, a new equilibrium system is formed between soil particles, ions, and water molecules. An external disturbance to the soil structure leads to a temporary reduction in soil strength, which subsequently recovers after a period of quiescence. The second mechanism is liquefaction damage. These two pathways to liquefaction—thixotropy-induced and liquefaction-induced damage—vary according to the clay content within the silt. A transition point exists with respect to clay content: for silty soils containing less than 9% clay, liquefaction is primarily associated with an increase in pore water pressure. Conversely, for silty soils with a clay content exceeding 9%, the thixotropic theory elucidates the observed liquefaction behavior.

In a comprehensive study, Xu et al. [14] focused on the high-salt silt found in the Yellow River Delta, conducting an extensive array of both in situ and laboratory mechanical tests on undisturbed samples. Employing a multidisciplinary approach that encompassed physical properties, chemical attributes, and microscopic analyses, the research provided a thorough examination of the mechanical properties and engineering characteristics of this specific silt. Building on these insights, cost-effective and environmentally sustainable additives were selected for further modification tests on Yellow River Delta silt. Subsequently, through a combination of experimental data and numerical analysis, the study elucidated the mechanical responses and deformation characteristics of the modified silt when used as a subgrade material.

Utilizing scanning electron microscopy, the study examined the microscopic composition and morphological characteristics of silt to gain insights into the development and constituents of its microstructural anisotropy [15]. The resolution gradually increased from (a) to (d), ranging from 100× to 1000×. Silt particles exhibit a variety of shapes, ranging from elongated strips and ellipses to sheet-like forms. This morphological diversity suggests that the spatial arrangement of these irregular particles likely varies across different orientations, thereby contributing to macroscopic anisotropy. Generally speaking, the long-axis direction of elongated particles tends to align preferentially perpendicular to the principal stress of greatest magnitude.

## 3. Physical and Chemical Properties

Currently, extensive research is being conducted both nationally and internationally on the physical and chemical properties of silt. This body of work encompasses a diverse array of topics including chemical composition, liquid plastic limits, particle composition, as well as permeability and capillary characteristics.

Silt is a fine-grained soil that consists mainly of fine-grained minerals such as kaolin, illite, and montmorillonite. These minerals have unique chemical properties that have a significant impact on soil structure and function. Montmorillonite, a 2:1 layered silicate mineral, is distinguished by its high cation exchange capacity and superior water adsorption capacity [16]. This makes montmorillonite valuable in soil improvement, wastewater treatment, and industrial applications. Kaolin is mainly composed of aluminum hydroxide and silicon oxide, which has high acidity and strong surface activity [17]. These properties make it widely used in the ceramics, paint, and cosmetics industries. Illite is structurally between kaolin and montmorillonite, with moderate cation exchange capacity and adsorption [18]. In addition, organic matter and microorganisms in silt soil can interact with these minerals, further affecting their chemical properties and soil function [19]. In order to better utilize and manage these resources, an in-depth study on the chemical properties of silt is essential [20].

In a focused study, Penman [21] sourced air-dried silt samples—comprising 90% silt, 10% sand, and 0% clay—from the drilling system at the Braehead Power Station site in Scotland. Utilizing a mud sampling method, soil samples with varying degrees of porosity (ranging from loose to medium-dense and dense) were prepared. Subsequent drained and undrained triaxial tests were conducted on these saturated silt samples to further our understanding of their mechanical properties.

Silt deposits, both recent and ancient, are prevalent across most regions of Alaska’s coastal continental shelf. These soils exhibit a varied gradation, ranging from pure silt to silty sand and clayey silt. In a seminal study, Wang [22] investigated Alaska’s salt-rich silt, comprising approximately 70–80% salt silt with a friction angle (φ) of 18 degrees. The findings indicate that, at low clay content levels, the silt behaves more akin to sandy soil rather than soft clay. Consequently, traditional empirical relationships linking strength, compressibility, and Atterberg limits are not readily applicable to this particular type of silt.

As offshore energy development advances in Alaska, the economic viability of constructing artificial islands has come into question, prompting the proposal of alternative offshore oil platform designs. Research indicates that the unique properties of Alaska’s seabed silt significantly influences both the design and construction of these offshore platforms. In an illuminating study, Börgesson [23] collected silt samples from boreholes extending through 6 m of ice and 15 m of subaqueous sediment beneath an oil platform in the Beaufort Sea. The study found that, similar to clay, Alaskan silt exhibits pronounced strength anisotropy. However, unlike clay, it demonstrates minimal creep strength loss, rendering the impact of creep on silt-based foundations less significant than on clay foundations. Despite the relatively low sensitivity of silt to disturbances, minimizing sample disturbance remains crucial for accurately assessing in situ silt strength, given its considerable effect on the undrained shear strength of the samples.

Brandon et al. [24] highlighted that the low-plastic silt from the Mississippi River exhibits distinct characteristics that set it apart from conventional soil types. Consequently, interpolation between sand and clay fails to offer an accurate methodology for characterizing this unique form of silt. Factors such as static pressure and vibrational forces are pivotal in determining the in situ density of silt, thereby underlining the need for specialized approaches in its analysis.

Capillary water absorption plays a crucial role in humidifying subgrade silt and serves as a significant mechanism for external water to infiltrate the soil structure of the subgrade. When the lower layers of subgrade soil are saturated by stagnant water in adjacent ditches, the upper layers remain relatively dry. Capillary action, or matrix suction within a defined range, facilitates the upward movement of water through the soil’s capillary pores. Conversely, once the surface soil of the subgrade slope becomes saturated, capillary forces also drive water horizontally into the subgrade.

Two key considerations must be addressed to understand the wetting of subgrade soil through capillary water absorption:

(1) The extent to which capillary water rises dictates the vertical zone of subgrade humidification.
(2) The term “capillary water absorption” is defined as the maximal mass of water absorbed per unit mass of soil when subjected to an adequate water supply. This parameter encapsulates the highest moisture content achievable via capillary water absorption in subgrade soil.

**Figure 2:**
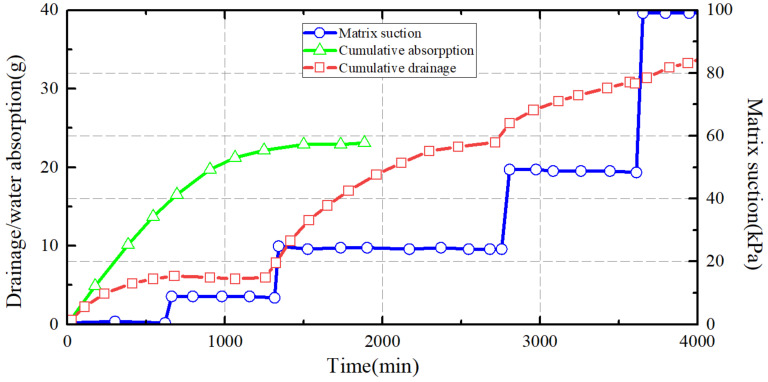
Cumulative drainage and cumulative water absorption curve of silt under different matric suction conditions.

Theoretically, capillary water absorption can be understood as follows: when the aggregate porosity of soil particles falls within a certain range, water in the pore channels forms a meniscus due to surface tension. This generates a pressure differential that leads to rapid liquid-level elevation, an effect substantially influenced by the soil’s state of compaction [25]. Figure 2 presents the cumulative drainage and water absorption curves for silt under varying conditions of base material suction.

**Figure 3:**
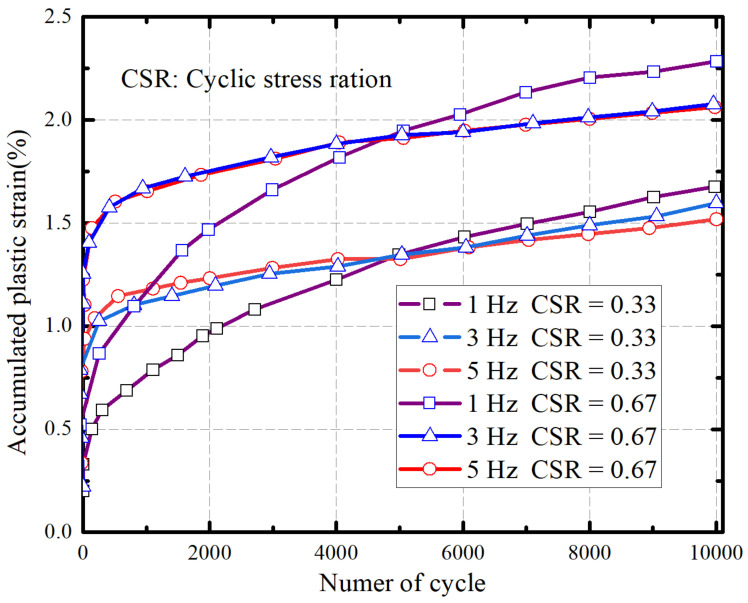
Accumulated plastic strain cyclic coefficient relationship curve of silt samples under different load frequencies.

In an extensive investigation into the engineering characteristics of silty soil, Ren [26] carried out an indoor large-scale dynamic triaxial test. Figure 3 shows the variation curve of the cumulative plastic strain of soil samples with cyclic oscillation at different load frequencies when the compaction degree is 94% and CSR is 0.33 and 0.67, respectively. It can be seen from the figure that the cumulative plastic strain of the sample develops rapidly in the early stage of loading, but the cumulative strain and growth rates are different. In the early stage of loading, under the same cyclic stress ratio, the greater the frequency of the specimen, the faster the growth rate of the cumulative plastic strain. After stabilization, the greater the frequency, the more stable the development trend of the cumulative plastic strain, and the larger the cyclic stress ratio, the more significant the phenomenon. In addition, the more frequently the specimen is subjected to loading throughout the test phase, the smaller the cumulative plastic strain variable. Similarly, Yuan et al. [27] observed that, in controlled indoor tests lasting 16 days, the silty soil remained either wet or excessively wet up to a height of 120 cm.

In order to investigate the impact of capillary water on the properties of silty sand in the Yellow River floodplain in East Henan, Yuan et al. [28] collected soil samples from the region and prepared them at compaction rates of 94%, 96%, and 98% to serve as control conditions. Various tests were conducted, including compaction tests, unconfined compressive strength tests, freeze–thaw tests, direct shear tests, and capillary water rise and control tests. Concurrently, a numerical simulation of the test data was performed to ascertain the functional relationship between the capillary water rise and the water absorption time. The results demonstrated that the capillary water rise height eventually stabilized over time; by the 10th day, it reached approximately 130 cm and peaked at 285 cm after stabilization. The speed of capillary water rise was found to be inversely proportional to soil compactness; at a 98% compactness level, both the capillary water pressure velocity and rising height were minimized. Similarly, the direct observation technique was employed to examine the capillary water rise behavior in the silty subgrade of the Yellow River floodplain [29]. The data revealed that the capillary water rise could achieve heights of over 70 cm within 10 days, after which the rate of rise gradually decelerated. The maximum height, 1.46 m, was attained after 66 days of stabilization. The influence of capillary water was particularly pronounced, especially when combined with rainwater infiltration from above and rising water levels from below. These conditions led to significant changes in the moisture content of the subgrade, potentially compromising its strength and long-term durability.

Based on the examination of the physical and chemical properties of silt, it has been observed that capillary water tends to rise to significant heights within this soil type. This rise in capillary water subsequently elevates the soil’s water content, which can lead to a degradation in road performance.

## 4. Mechanical Properties

**Figure 4:**
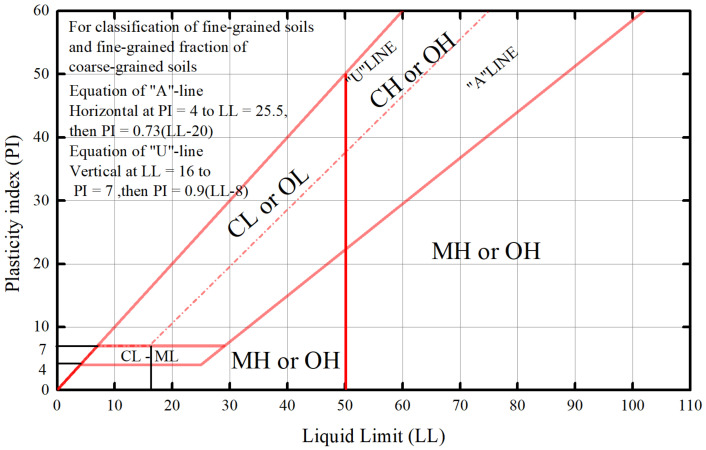
Soil classification plasticity chart.

Intermediate soil serves as a transitional soil type between sand and clay. According to the soil classification plasticity chart [30], as shown in Figure 4, such soils are categorized as CL-ML, ML, or OL, falling below Line A and to the left of Line B. In domestic classifications, these are typically referred to as sub-sandy soils or sub-clays containing sand or silt. Despite their fine-grained nature and retention of sandy characteristics, these soils have received limited attention in the realm of engineering mechanics. They often exhibit low plasticity, gap grading, and poorly defined dynamic properties. This category encompasses not only natural silt, sandy cohesive soil, and organic soil, but also various modified soils such as special mineral-modified liquefiable mixed soils and rubber granular cohesive soils, which share similar dynamic characteristics. Hence, they are considered to be an intermediate medium within the spectrum of fine-grained soils.

Recent research has increasingly focused on intermediate soil’s dynamic characteristics and liquefaction mechanisms, driven by the distinct roles played by the two types of granular materials present in the soil and their unique inter-particle interactions. Furthermore, the microscopic study of fine particles in intermediate soil lays the foundation for broader investigations into the dynamic microscopic properties of fine-grained soils.

The study of the dynamic microstructure of natural intermediate soils has largely focused on liquefaction identification and failure mechanisms. For instance, Boulanger and Idriss [31,32] pioneered the classification of intermediate soils into two categories: those exhibiting “sandy behavior” and those displaying “clay-like behavior,” using a plasticity index (Ip) of 7 as the demarcation point. According to this classification, soils in the former category experience stress or strain failure, while those in the latter undergo cyclic softening failure. Bray and Sancio [33] conducted tests to analyze the dynamic performance of typical intermediate soils, substituting the plasticity index of clay minerals for the traditional measure of clay content percentage to determine liquefaction sensitivity. Salam and Bakirl [34] employed the slurry deposition method to perform uniaxial compression and dynamic torsional shear triaxial tests on remolded low-plasticity silt within the intermediate category, exploring various factors that influence dynamic strength. Similar research endeavors include Wijewickreme’s team’s work on liquefaction discrimination studies for diverse tailings [35,36] and river sediments [37].

Deng [38] studied the microstructure of loess in different regions and soil environments, conducted an energy spectrum analysis on high-power electron microscope scanning images, and conducted earthquake settlement experiments and numerical simulations. The microstructure characteristics of loess under different soil-forming environments, the microstructure mechanism and dynamic constitutive relationship of loess seismic subsidence, and the ground motion characteristics of loess sites were studied. A microstructure evaluation method for loess seismic subsidence and a microstructure acid improvement technology for loess foundation seismic subsidence were proposed.

In a specific state of pore pressure, an adsorption equilibrium exists between the pore spaces and soil particles, leading to a deduced seismic subsidence equation in terms of the pore ratio. The seismic subsidence in loess is quantified as the sum of the compression in overhead pores and the disintegration of cementitious aggregates. Employing electron microscope imagery, the area of these overhead pores can be calculated to determine the microscale seismic subsidence coefficient [39,40,41]. These findings align with the loess seismic subsidence deformation model proposed by Shen and Li [42]. This model identifies three distinct stages under low stress: elastic deformation, plastic deformation via pore-filling, and compaction deformation, representing a typical strain-hardening behavior. Conversely, under high-stress conditions, shear collapse deformation exhibits strain-softening behavior, progressing through cementation fracture softening and yield deformation. Loess, as a representative intermediate soil, exhibits less pronounced dynamic strain-hardening compared to sand, and less dynamic strain-softening compared to clay. The cohesion among clay particles is robust, allowing for minimal particle dislocation and a swift transition to the softening phase without undergoing a hardening process. In a related study, Zhang and Gao [43] conducted dry soil-layered compaction tests on remolded hollow cylinder samples of silt, controlling for dry density. They simulated the pore water pressure variations in saturated silt under wave loading and posited that the development of pore water pressure in silt follows a “rapid–stable–rapid” growth trajectory.

Silt, classified as an intermediate medium, is prone to sudden collapse failure [44]. Prior to the precursor of collapse, a connected stress zone is formed; within this zone, the continuous dislocation of particles results in a gradual tightening of the hysteresis loop shape. This alteration in energy serves as the internal mechanism that triggers the abrupt failure. To further investigate this phenomenon, the Particle Flow Code (PFC) discrete element method is employed to simulate the stress-controlled dynamic triaxial correlation process.

**Figure 5:**
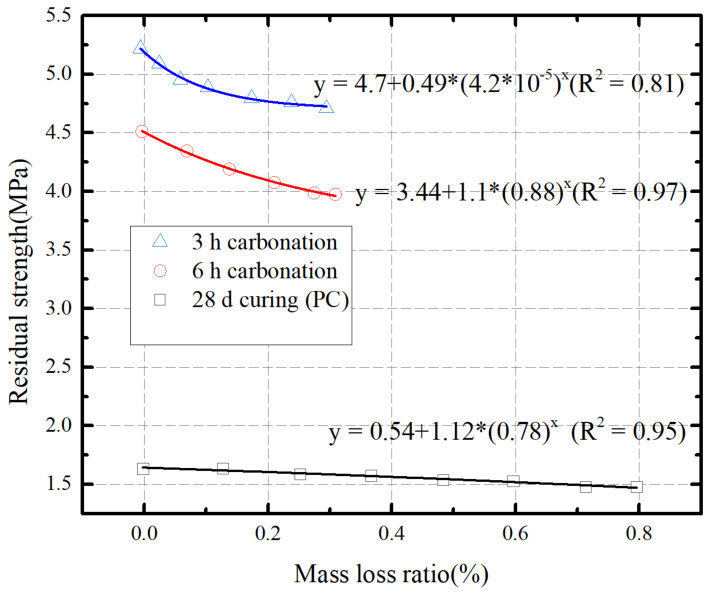
Relationship between residual strength and mass loss ratio of MgO-carbonated and PC-stabilized silt after freezing–thawing cycles.

Jing [45] conducted a qualitative investigation into the liquefaction of site silt under K0 consolidation conditions, utilizing small-scale shaking table tests to validate the applicability of the pore water pressure growth model. The test’s approximate similarity law was derived from the motion’s differential equation and was subsequently applied in the test analysis. Figure 5 illustrates the relationship between the residual strength and the mass loss ratio of the carbonated/stabilized samples after freeze–thaw cycles. The residual strength decreases with the increase in mass loss rate. The relationship between residual strength and mass loss rate can be suitably represented by an exponential function, with a significant correlation coefficient [46].

Prior et al. [47] conducted indoor vibration tests to examine the dynamics of soil strength loss and recovery, revealing that the mechanical properties of silty soil from the Yellow River estuary are influenced by the extent of inter-particle contact. Drumm et al. [48] established that, under constant confining pressure, an inverse relationship exists between the viscoelastic dynamic modulus and the changes in differential stress along the shaft. They noted that as differential stress increases, the dynamic modulus tends to decrease within a specific range; however, this rate of decline decelerates when the differential stress reaches elevated levels. Li et al. [49] investigated the compaction curves of fine-grained soil, identifying distinct characteristics and fitting the curve transitions from dry to wet conditions. They derived clusters of compaction curves under varying pressure regimes, assuming known soil properties. Yang et al. [50] examined the stability of silty sand mixtures, focusing on how performance remained constant despite variations in fine particle content. They discovered that the degree of variation in slope performance increased with the rise in relative density. Furthermore, the study concluded that the relationship between the observed values and the porosity ratio could be adequately modeled by a standard curve when the total fine-mixture stress reached its maximum value.

Through comprehensive testing, Seed and Chan [51] discovered that sandy clay exhibits distinct stress–strain relationships depending on whether it is prepared by kneading or static compaction, even when the density and moisture content are held constant. Papadimitriou et al. [52] observed that the stress–strain and volumetric behaviors of Toyoura sand are influenced by the method of preparation, specifically highlighting four techniques: dry loading deposition, wet loading compaction, the sand rain method, and dry loading compaction. Delage et al. [9] conducted mercury intrusion tests on silt samples with varying water contents and investigated the correlation between water content and sample porosity. Magistris and Tatsuoka [53] demonstrated that the effect of water content on stress changes in Metamo silty soil is primarily limited to stages with minor alterations, under consistent testing conditions, and that its impact on shear force can generally be disregarded. T. W. Lambe [54] ascertained that clay exhibits a flocculated structure when the water content is below the optimum level, and a pronounced directional alignment when it surpasses this level. Similarly, Seed and Chan [51] studied silt samples with a wide range of water contents and found that wetter soil samples tend to be more dispersed.

## 5. Silt Improvement Technology

In the early 20th century, Western nations initiated research into soil stabilization techniques. However, until the 1960s, the focus was predominantly on using inorganic binders for stabilization, which chiefly included cement, quicklime, and fly ash. Beginning in the 1970s, advanced research on soil stabilizers was conducted in Western countries, Japan, Australia, and elsewhere, leading to a range of commercially available soil-stabilizing products and corresponding enterprises, such as Roadbond in the United States and UKC in Japan. Subsequent to these developments, international scholars have engaged in extensive experimental research on soil stabilization [55]. For instance, laterite soil stabilized with 5% phosphoric acid exhibited a strength of 40 MPa after a curing period of 28 days. Bel [56,57] employed a combination of pulverized fuel ash (PFA), cement, and lime to fortify clay. Atlm and Al-Sharif1 [58] investigated the efficacy of burnt olive waste as a soil stabilizer. Additionally, Byung Sik Chun et al. [59] examined the outcomes of using a mixture of fly ash, gypsum, and cement (FGC) for soil reinforcement.

Currently, a wide array of soil stabilizing agents have been developed on the international stage. In the United States, notable products include ROADBOND, SOIL ROC, EARTAZYME, and BASE-SEAL. Canada has contributed with brands such as CBR-PLUS, B.C. Stabilizer, and Roadpacker Plus. South Africa offers ISS and SUPERSOIL, while Australia has developed AUSTWIDE. In Europe, Greece features Eeo Crete, and the Netherlands has introduced Frisol and Probase. Additionally, South Korea has contributed with its Condor SS. These products collectively exemplify the global advancements in soil stabilization technologies.

The advancement of soil stabilization technology in China has been relatively recent, commencing in earnest in the 1990s. During this period, several domestic institutions began importing high-tech, high-performance soil stabilizers from Japan. Subsequent research and development efforts have led to the localization of this technology. Notable examples include the NCS clay curing agent, co-developed by Beijing Autoset and the Highway Science Research Institute of the Ministry of Communications; the ZDYT soft soil curing agent by Zhejiang University; SH polymer material curing agents by Lanzhou University; and both the SEU liquid and SEU-2 silt curing agents by Southeast University. Additionally, the Wuhan University of Technology has produced the HS curing agent, while Shanghai Kexin Building Materials Co., Ltd. has introduced the NG and LY curing agents. Despite these advancements, there has been a limited focus on soil consolidation agents specifically designed for silt. Zhu et al. [60] have made contributions in this area by conducting both unconsolidated undrained triaxial shear tests (UU) and consolidated undrained triaxial shear tests (CU) to examine the strength and deformation characteristics of soil stabilized with varying combinations of lime, cement, and SEU-2 curing agents.

**Figure 6:**
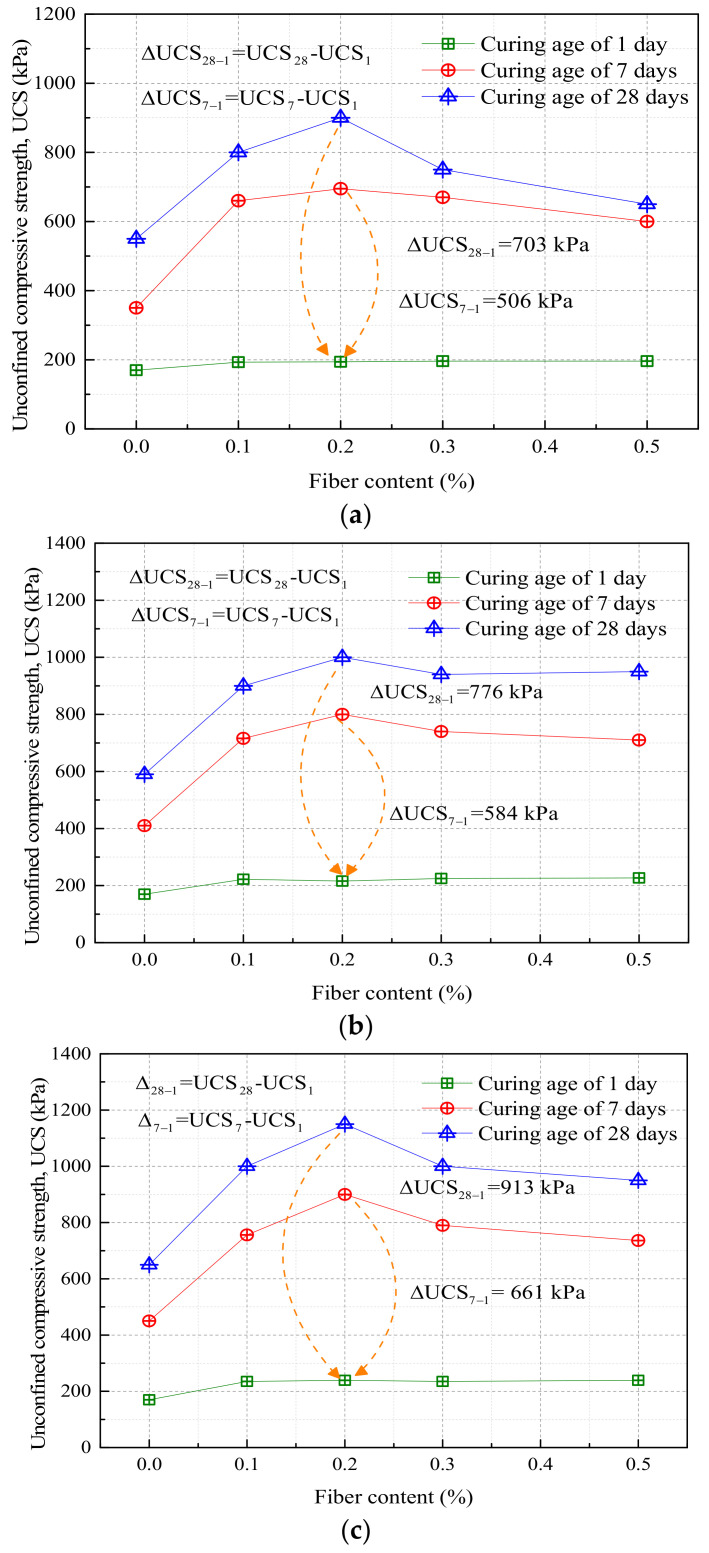
Change in unconfined compressive strength of improved soil under different degrees of compaction: (**a**) compaction degrees of 94%; (**b**) compaction degrees of 96%; (**c**) compaction degrees of 98%.

Figure 6 illustrates the variations in unconfined compressive strength of improved silty soil across different compaction degrees (94%, 96%, and 98%) [61]. Evidently, the unconfined compressive strength of the modified silty soil enhances with an increment in fiber content, reaching its pinnacle at a fiber content of 0.2%. Beyond this fiber content, the strength of the improved soil gradually diminishes and stabilizes. Concurrently, with an increase in fiber content, the failure mode progressively transitions from “brittle failure” to “ductile failure”. At a curing age of one day, the strengths of the improved silty soil, regardless of the fiber content, exhibit negligible variation, aligning with the strength of the simply improved silty soil (170 kPa). At a curing age of seven days, the strength of the improved silty soil containing 0.2% fiber achieves 695 kPa at a 94% compaction degree; at a curing age of 28 days, it experiences an augmentation of approximately 362% at a 96% compaction degree. Moreover, with an uptick in compaction degree, the strength discrepancy in improved silty soil progressively enlarges, reaching maximum differences of 913 kPa and 661 kPa, respectively.

In silt reinforcement applications, commonly used materials such as cement and quicklime are often employed as reinforcing agents, which can be applied through either single or dual mixing methods. For instance, Wang et al. [62] conducted water stability tests using slag powder and quicklime as reinforcing agents for silt. The results demonstrated that slag powder outperformed quicklime in enhancing silt stability. Mojtahedi et al. [63] executed a comprehensive set of tests to explore how various parameters, such as cement content, water–cement ratio, and age, affect the performance characteristics of cement-stabilized soil. The findings indicated a negative correlation between the water–cement ratio and the unconfined compressive strength of the cement-stabilized soil when the cement content was held constant. Conversely, the unconfined compressive strength increased with rising cement content when the water–cement ratio remained the same. Yang [64] conducted tests comparing the efficacy of cement lime and water–glass–lime mixtures. The results revealed that, with equal lime contents, mixtures containing cement lime exhibited superior performance compared to those with water–glass–lime.

In the current landscape, where advancements in materials science and technological innovation are rapid, there is an increasing public awareness of environmental sustainability. Consequently, international research focused on new materials, such as fibers and surfactants, for applications in the construction industry and soil improvement has deepened. The failure mechanisms and stress–strain characteristics of polymer-modified silt were investigated [65]. Various types of triaxial tests, including unconsolidated undrained (UU), consolidated undrained (CU), and consolidated drained (CD), were conducted on samples of silt modified with polymers. The results indicated that polyacrylamide (PAM) enhances both the compressive deformation and shear resistance of silt. Conversely, silt modified with Sorran sodium silicate showed improved shear resistance but reduced compressive deformation. A significant finding was that, despite these improvements, both modifications exhibited subpar water resistance. This outcome, while revealing areas for further optimization, provides a crucial foundation for addressing the challenges of stability and deformation in subgrade engineering projects located in silt-prone areas.

**Figure 7:**
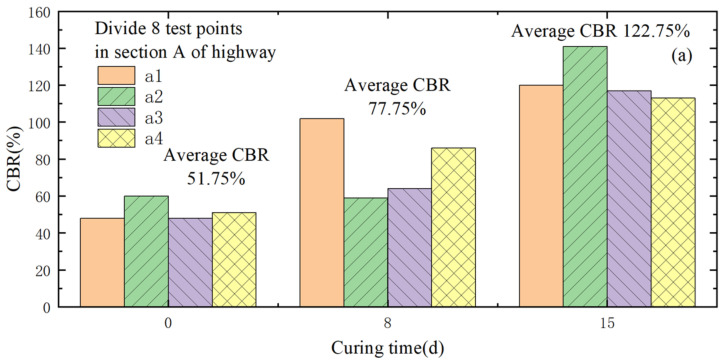

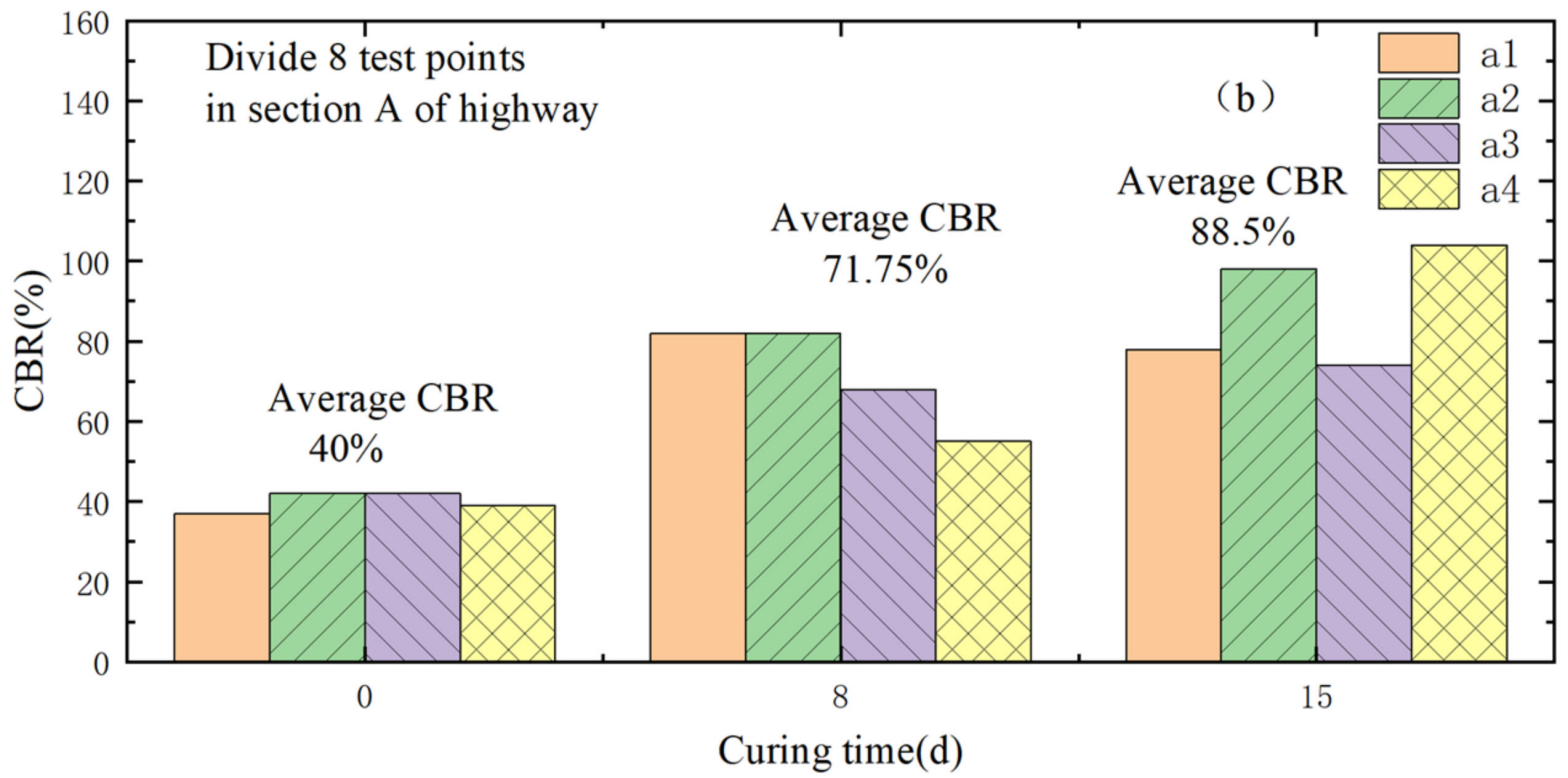
Effect of curing time on the CBR of stabilized silt: (**a**) Test section A, 12% lignin-stabilized silt; (**b**) Test section B, 8% lignin-stabilized silt.

Experiments were conducted to ascertain the feasibility of using lignin-stabilized silt for highway subgrades [66,67,68]. Figure 7 shows the effect of curing time on the CBR of stabilized silt. The findings indicate that silt solidified with 2% lignin exhibits superior mechanical properties compared to silt solidified with 8% quicklime. Additionally, a comprehensive analysis was carried out on the micro-mechanisms and thermodynamic properties of the lignin-modified silt. The results revealed that both the performance and thermal resistance coefficient of the lignin-stabilized soil improve as the lignin content increases, reaching optimal levels at a 12% concentration. This enhancement is attributed to the presence of cementitious substances in the microstructure, which contribute to the compaction of the silt.

Wang et al. [69] conducted experiments to evaluate the impact of active MgO-enhanced silt on permeability coefficients. Using a control group for comparison, the results demonstrate that the soil improved with active MgO exhibits similar impermeability characteristics to soil improved with cement. Moreover, the impermeability improves as the active MgO content increases, while permeability decreases. In a parallel study, Cai et al. [70] investigated the microstructure of activated magnesia-carbonated silt. The research revealed that upon carbonation, both the magnesia material and the silt particles exhibited a condensation phenomenon, thereby improving the degree of compaction.

The magnesium oxide had a positive effect on the process of improving the physicochemical properties of soil. This phenomenon can be explained from the following perspectives:

(1) Chemical Neutralization: Magnesium oxide (MgO) can be dissolved in water to produce magnesium ions (Mg^2^⁺) and hydroxide ions (OH^−^). When these ions react with acidic components present in the sludge, they can undergo a neutralization reaction, elevating the pH of the sludge. This pH increase can inhibit the growth of certain harmful microorganisms and enhance the activity of beneficial ones, further optimizing sludge treatment.
(2) Flocculation Effect: Magnesium ions can bridge with negatively charged particles in the sludge, an action termed flocculation. This process facilitates the aggregation of tiny particles in the sludge into larger ones, making the sludge easier to settle, dewater, and separate.
(3) Enhancing Biological Treatment: Certain microorganisms that are essential for sludge treatment require magnesium ions for their growth and activity. Therefore, the addition of magnesium oxide might supply the necessary magnesium ions, enhancing the efficiency of the biological treatment process.
(4) Inhibition of Harmful Gas Production: Magnesium oxide can suppress the production of certain harmful gases, like hydrogen sulfide (H_2_S), during sludge processing. This is due to magnesium ions reacting with sulfide ions in the sludge to form insoluble magnesium sulfide salts, reducing the release of H_2_S.
(5) Thermal Stability Enhancement: For sludges that need heat treatment, the addition of magnesium oxide can enhance their thermal stability, reducing the decomposition of organic materials and the release of harmful substances at high temperatures.

**Figure 8:**
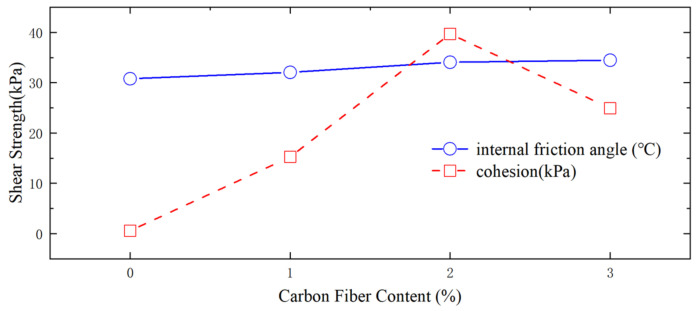
Effect of carbon fiber on shear strength parameters.

**Figure 9:**
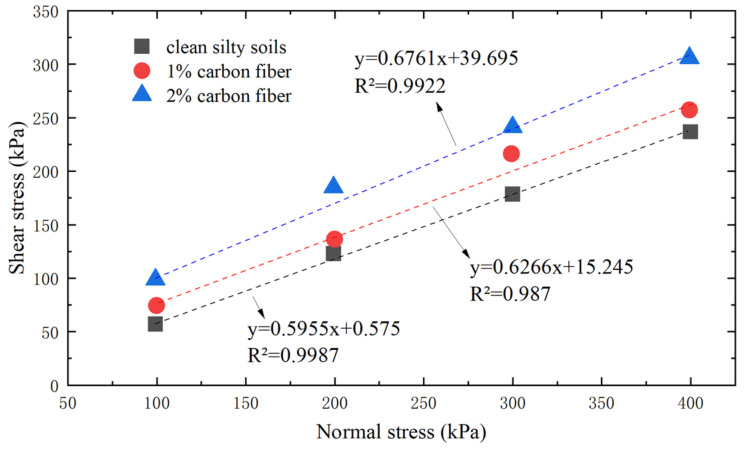
Relationship between shear strength and normal pressure under different carbon fiber content conditions.

The influence of carbon fibers and nano-silica on the shear resistance of silty soil was examined [71]. The results of the experiment revealed that carbon fibers had a notably positive impact on both the internal friction angle and cohesive properties of the silty soil, as depicted in Figure 8. Conversely, nano-silica was found to enhance only cohesive performance, as illustrated in Figure 9.

In a study conducted by Durczak [72], the effects of two types of fibers, differing in length and content, on the seepage rate and seepage force of silt were investigated. The results of the experiment demonstrates the controllability of seepage rate and seepage force in reinforced silt. Furthermore, based on these test findings, a regression model was developed to ascertain seepage velocity and permeability. Additionally, the study explored the impact of basalt fiber length, content, and compaction on the physical and mechanical properties of silt. Notably, the tests revealed that fiber reinforcement enhances the shear resistance of silt while reducing its compressibility and permeability.

Silty soils, characterized by their specific particle sizes and structures, typically possess lower shear strength and stability. This can pose challenges in many civil engineering projects, especially in road and infrastructure construction. To enhance the engineering characteristics of silty soils, researchers and engineers have begun to explore the incorporation of recycled polyester fibers as a reinforcing agent.

When mixed with silty soils, these fibers can form a uniformly distributed three-dimensional network, contributing to an improvement in the overall strength of the soil matrix. The fibers not only boost the tensile and shear properties of the soil but also, owing to their robust weather resistance, maintain these enhanced characteristics over extended periods, even under adverse environmental conditions. Additionally, utilizing recycled fibers aligns with sustainability goals by reducing landfill waste and environmental contamination, as they are manufactured from repurposed materials.

However, several challenges remain to be addressed. Determining the optimal fiber content, length, and dispersion methodology is crucial for achieving the best reinforcement outcomes. Moreover, while recycled polyester fibers are eco-friendly, potential environmental impacts stemming from their production and application warrant further study. In conclusion, recycled polyester fibers present significant promise as a sustainable reinforcement agent in silty soils. As research progresses, we can anticipate a broader application of this material in future civil engineering endeavors.

## 6. Conclusions and Prospect

By comprehensively reviewing recent advancements in the research concerning the evolution of the engineering characteristics and reinforcement technologies in subgrade silt, the following conclusions can be derived:

(1) Soil reinforcement improves the engineering properties of subgrade soil. However, it does not notably enhance the soil’s crack resistance, leading to possible road-surface cracks. This reinforced soil also shows limited resistance to piping, liquefaction under vibrational loads, and often results in brittle failure.
(2) Using cement lime for soil modification can reduce its water retention and nutrient content, threatening nearby plant life and affecting the durability of underground structures. Both cement and lime soils show brittleness, which can compromise the stability of foundational structures under specific loads.
(3) Fibers have proven to enhance the strength of sand and clay. Yet there is a research gap concerning their effects on silt, especially about changes in physical and mechanical characteristics, durability, and heat conduction properties. Understanding these effects is crucial for the effective reinforcement of silt with fibers.
(4) In line with global sustainability efforts, it is essential to focus on developing silt reinforcement methods that are both efficient and environmentally friendly.

The reinforcement of silty roadbeds confronts several specific challenges in the foreseeable future. Firstly, with the escalating impacts of global climate change, extreme weather conditions such as heavy rainfall, droughts, and freeze–thaw cycles could adversely affect the stability of silty roadbeds. Therefore, reinforcement techniques need to evolve to offer heightened adaptability and resilience to these changes. Secondly, with the rapid pace of urbanization, land resources are becoming increasingly scarce. This intensifies the importance of judicious site selection and efficient resource utilization during construction. Furthermore, as innovative reinforcement materials and techniques continuously emerge, a core concern becomes ensuring their long-term performance, reliability, and cost-effectiveness in comparison to or exceeding traditional methods. Additionally, in an era increasingly conscious of environmental protection, realizing green and sustainable reinforcement approaches—aimed at reducing carbon emissions and other environmental pollutants—will be a pivotal challenge faced in the domain of silty roadbed reinforcement.

## Data Availability

Not applicable.
